# Effect of breastfeeding promotion interventions on breastfeeding rates, with special focus on developing countries

**DOI:** 10.1186/1471-2458-11-S3-S24

**Published:** 2011-04-13

**Authors:** Aamer Imdad, Mohammad Yawar Yakoob, Zulfiqar A Bhutta

**Affiliations:** 1 Division of Women & Child Health, The Aga Khan University, Karachi, Pakistan

## Abstract

**Background:**

Given the recognized benefits of breastfeeding for the health of the mother and infants, the World Health Organization (WHO) recommends exclusive breastfeeding (EBF) for the first six months of life. However, the prevalence of EBF is low globally in many of the developing and developed countries around the world. There is much interest in the effectiveness of breastfeeding promotion interventions on breastfeeding rates in early infancy.

**Methods:**

A systematic literature was conducted to identify all studies that evaluated the impact of breastfeeding promotional strategies on any breastfeeding and EBF rates at 4-6 weeks and at 6 months. Data were abstracted into a standard excel sheet by two authors. Meta-analyses were performed with different sub-group analyses. The overall evidence were graded according to the Child Health Epidemiology Reference Group (CHERG) rules using the adapted Grading of Recommendations, Assessment, Development and Evaluation (GRADE) criteria and recommendations made from developing country studies for inclusion into the Live Saved Tool (LiST) model.

**Results:**

After reviewing 968 abstracts, 268 studies were selected for potential inclusion, of which 53 randomized and quasi-randomized controlled trials were selected for full abstraction. Thirty two studies gave the outcome of EBF at 4-6 weeks postpartum. There was a statistically significant 43% increase in this outcome, with 89% and 20% significant increases in developing and developed countries respectively. Fifteen studies reported EBF outcomes at 6 months. There was an overall 137% increase, with a significant 6 times increase in EBF in developing countries, compared to 1.3 folds increase in developed country studies. Further sub-group analyses proved that prenatal counseling had a significant impact on breastfeeding outcomes at 4-6 weeks, while both prenatal and postnatal counseling were important for EBF at 6 months.

**Conclusion:**

Breastfeeding promotion interventions increased exclusive and any breastfeeding rates at 4-6 weeks and at 6 months. A relatively greater impact of these interventions was seen in developing countries with 1.89 and 6 folds increase in EBF rates at 4-6 weeks and at 6 months respectively.

## Background

Breast-milk provides numerous immunologic, psychologic, social, economic and environmental benefits; and is a natural first food and an ideal nutrition for the newborn [1]. Breastfeeding is therefore recommended as the optimal strategy for feeding newborns and young infants [2-5]. Breast-milk has a significant positive impact on child growth and development and decreases the risk for many acute and chronic diseases [6-11], including infections such as diarrhea and respiratory tract infections during infancy [12]. It also confers benefits on the mother such as reduced postpartum bleeding and early uterine involution, coupled with decreased risk of breast and ovarian cancers and hip fractures later in life [13]. The WHO recommends exclusively breastfeeding the infant for the first six months of life to achieve optimal growth, development and health [14]. Thereafter, appropriate complementary foods should be introduced, while breastfeeding continued up to two years of age or beyond. Nevertheless, EBF remains uncommon in most countries (both developed and developing), even in countries with high rates of breastfeeding initiation [15,16]. EBF rates in infants less than six months of age varied from as low as 20% in central and eastern European countries to 44% in South Asia [17]. The reasons for low prevalence of EBF could be lack of support for breastfeeding by social workers and health care providers, emotional stress in mothers and their perception of not having enough breast milk, and pressure from close relatives to introduce other liquid and solid foods, unsupportive hospital practices that delay early initiation of BF, maternal employment, and lack of commercial advertising [18]. For reasons like these, effective programs of education, counseling and support are considered necessary to not only promote breastfeeding but also to prolong the duration of EBF to up to six months of age. Breastfeeding promotion, therefore, is a global priority with benefits for maternal and child health, especially in low-/middle-income countries where its relevance for child survival is undisputed [19].

EBF for six months might be difficult, particularly where maternal malnutrition is common [20]. Inadequate assistance to mothers wishing to breastfeed has contributed to the unacceptably high rate of premature cessation of breastfeeding [21]. Education and support is therefore the cornerstone, supporting the framework of lactation and breastfeeding [22]. Two main strategies to promote EBF include The Baby Friendly Hospital Initiative and secondly, the use of peer counselors, especially in settings where most babies are delivered at home. Comprehensive and culturally appropriate breastfeeding education through counselors (be they doctors, nurses, midwives, lactation consultants or peer counselors) during the prenatal period, in the hospital during first week postpartum, and repeated, continual support in the mother’s home may be critical for facilitating breastfeeding among mothers, especially those belonging to the low-income groups [23-25]. Both prenatal and postnatal education is important as the incidence of breastfeeding is affected primarily by prenatal education, whereas the duration and exclusivity of breastfeeding is affected by both prenatal and postpartum management [26,27]. Evidence is also available on the beneficial effect of social support on health during pregnancy and labor and in encouraging successful breastfeeding [28-30]. Peer counselors and community health workers can be an important source of breastfeeding support; for example, in promoting positive outcomes, increasing breastfeeding rates, increasing maternal satisfaction, and decreasing infant hospital readmission rates [31]. Other strategies to protect breastfeeding include the International Code of Marketing of Breast-milk Substitutes and communications campaigns.

The Cochrane review [32] on the subject includes only those studies which provided support to breastfeeding mothers and studies with other promotional interventions where education was the primary intervention were excluded. Our review incorporates both education and support studies, includes studies published after this review and also applies the Child Health Epidemiology Reference Group (CHERG) guidelines for inclusion of estimates into the Lives Saved Tool (LiST) model [33], especially on studies from developing countries. A prized addition to this review is separate sub-group analyses with respect to geographical location (developed and developing), timing of intervention (prenatal, postnatal or both), mode of delivery (individual or group counseling), level of care (community, facility or both) and individual components of breastfeeding promotion intervention (education alone, lay support, professional support, etc.). Our aim in this review was to evaluate all studies which investigated the impact of breastfeeding promotion interventions on exclusive and any breastfeeding rates at 4-6 weeks and at 6 months.

## Methodology

### Search strategy

Literature search was carried out to identify studies that evaluated the impact of breastfeeding education/support on breastfeeding rates. The search was conducted up to October 2010. The databases searched were PubMed, Cochrane Database of Systematic Reviews and regional WHO databases. Besides, hand search of bibliographies of relevant reviews was performed, experts in the field were contacted for further data or for unpublished trials. The search strategy used was:

("Breast Feeding"[Mesh] OR breastfeed* OR lactation) AND (education OR promotion OR counseling OR intervention OR support OR Social Support)

For preliminary selection, no restriction was made with respect to the language of the article. However, non-English articles were not translated and if the desired information was available in the abstracts then that was used; otherwise, the article excluded.

### Selection

All studies that were included in our review looked at patterns of breastfeeding at 4-6 weeks and at six months postpartum.

#### Inclusion criteria

• The study designs selected were randomized and quasi-randomized trials (studies that lacked true randomization and where methods of sequence generation were not adequate).

• The studies selected were from both developed and developing countries.

• The intervention included breastfeeding education and/or additional support given to mothers through counselors (be they doctors, nurses, midwives, lactation consultants or peer counselors) in individual or group sessions. Individual support included both face to face and via Telephone.

• The studies were included irrespective of the mode of delivery, whether vaginal or cesarean.

• All studies were included irrespective of language. For non-English articles, we primarily relied on the abstracts but did not translate the entire article into English. If the desired outcome was not present in the abstract, then the study was excluded.

• All studies where intervention (education/support) was given either in prenatal, postnatal, or combined prenatal and postnatal periods, were included.

• All studies were included in the meta-analyses irrespective of the lost to follow-up percentages.

#### Exclusion criteria

• All studies dealing with web- or internet-based interventions were excluded.

• All studies in which interventions were given to preterm/very preterm babies and low birth weight/very low birth weight babies were excluded.

• All studies in which the education or support was given primarily to fathers or to other family members like grandparents were excluded.

• Other interventions for promotion of breastfeeding like skin-to-skin contact or delayed pacifier use or motivational interviews with the goal of decreasing ambivalence and resistance toward sustained breastfeeding were excluded. Similarly, studies where breastfeeding education was provided in the form of a package with other interventions were also excluded.

• All intervention controlled trials that were not randomized or quasi-randomized were excluded. Similarly, before-after study designs, cohort and cross-sectional studies were excluded.

Breastfeeding interventions included in our review involved 1) formal or structured breastfeeding education (Ed) defined as one-to-one or group education sessions or classes (e.g., curriculum or standard agenda) directed at mothers or other family members. 2) professional support divided into system level support (PS-SL) involving interventions at mass level like implementing policies of baby-friendly hospital initiative (BFHI) or training of health professionals; and individual level (PS-IL) where support was provided individually to mothers during hospital stay or outpatient clinics; social support (e.g., home visits or telephone support) from health professionals]; and 3) lay support (LS) in which there was social support (e.g., home visits or telephone support) from peers. These categories of interventions are not mutually exclusive and may overlap. The outcomes considered in our review included exclusive and any breastfeeding rates at 4-6 weeks and at six months postpartum. EBF was defined as child receiving only breast milk and no other type of milk or solids but allows for vitamins, drops of other medicines and oral rehydration therapy. Any breastfeeding comprised of breast milk given either alone, with formula milk and/or solids.

### Data abstraction and validity assessment

The data were extracted by two researchers into a standard web excel sheet prepared by the CHERG methods group [33]. The variables considered included, for example, location of the study, setting, study design, blinding assessment, allocation concealment, intention-to-treat analysis, lost to follow-up rates, intervention and control group definitions and study limitations. Each study was graded according to GRADE criteria [34]. In this method of qualitative evaluation, all RCTs received an initial score of ‘high’ and an observational study as ‘low’. The study scores were adjusted depending on limitations of the study design. Trials with a final grade of ‘high’ or ‘moderate’ and ‘low grade’ were included in the analysis with exclusion of studies with a final grade of ‘very low’. The overall quality of evidence of an outcome was based on three components: 1) the volume and consistency of the evidence; 2) precision of the effect; and 3) directness [33,34]. The studies from developing countries were used to recommend estimates for inclusion into the Live Saved Tool (LiST) model. In that model, increases in coverage of an intervention result in a reduction of cause-specific deaths, reduction of a risk factor or, using this paper for example, increase in breastfeeding rates. For more details of the review methods, the adapted GRADE approach or the LiST model see the CHERG method’s summary [33].

### Quantitative data synthesis

We generated meta-analyses using RevMan 5.0 software for outcomes where more than one study was available [35]. The assessment of statistical heterogeneity among trials was done by visual inspection i.e. the overlap of the confidence intervals among the studies, and by the Chi square (P-value) of heterogeneity in the meta-analyses. A low P value (less than 0.10) or a large chi-squared statistic relative to its degree of freedom was considered as providing evidence of heterogeneity. The I^2^ values were also looked into, and an I^2^ greater than 50% was taken to represent substantial and high heterogeneity. In situations of substantial or high heterogeneity being present, causes were explored and random effects model was used and although, this random model is not a substitute for a thorough investigation of heterogeneity, it was primarily to take into account heterogeneity that could not be explained. The summary estimates were presented as Relative Risk (RR) with 95% confidence intervals (CI). In order to avoid publication bias, we also contacted experts in this field to share data on their studies that may have remained unpublished. For factorial designs, we used two data sets from each study, assuming a significant interaction between the two treatment groups. For cluster randomized trials, the cluster-adjusted values were used if reported in the studies themselves. We planned to do subgroup analyses for studies; conducted in developed countries vs. developing countries; group vs. individual counseling; timing of intervention (pre-natal, postnatal vs. prenatal and postnatal) and community based facilitation vs. facility based counseling. The objective of these stratifications was to assess the effectiveness of elements like availability of facilities. For example a lot of deliveries in developing countries occur at home and facility based strategies would not be effective in these scenarios. The World Bank list of economies (July 2009) was used to classify countries into developing and developed [36]. Low- and middle-income countries were taken as developing, while high income countries were taken as developed.

## Results

### Trial flow and study characteristics

**Figure 1:**
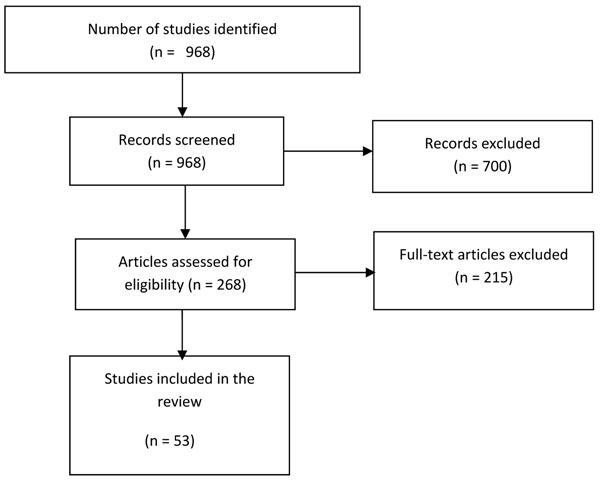
Flow diagram showing identification of studies

Figure 1 shows the search flow diagram. The search strategy used generated 968 titles/abstracts, which were screened and 268 abstracts were preliminarily selected with potential of inclusion in our review. We thoroughly reviewed the abstracts and full texts, where available, of these and finally, 53 studies met our inclusion criteria. The remaining 213 studies were excluded. Additional File 1 outlines the characteristics of included studies.

### Quantitative data synthesis

#### EBF rates at 4-6 weeks

There were thirty two randomized and quasi-randomized controlled trials that gave results of breastfeeding promotion interventions on EBF rate at 4-6 weeks postpartum [18,25,37-67], of which ten were developing country studies [18,25,37-42,64,66]. There was a statistically significant 43% increase in EBF rate at 4-6 weeks (RR = 1.43; 95% CI: 1.28 – 1.60), with 89% and 20% significant increase in developing and developed countries, respectively (Additional File 2A). Sub-group analyses according to timing of intervention showed that prenatal, postnatal and combined all had statistically significant impacts, with the highest impact being that of prenatal counseling (Additional File 3A). Education, professional support and lay support each alone had significant impacts (Additional File 4A). Group counseling had a greater impact (67% increase in EBF rate at 4-6 weeks), compared to individual counseling (38% increase) (Additional File 5A). The results were statistically significant at all levels of care (community, facility and both combined) (Additional File 6A).

#### EBF at 6 months

Fifteen studies were found that looked at this outcome [31,38,50,52,54,60,64,66,68-74], of which six were from developing countries [38,64,66,68-70]. There was an overall 137% increase in EBF rate with promotion interventions, with a significant 6 times increased incidence in developing countries, compared to 1.3 times in developed countries (Additional File 2B). The sub-group analysis with respect to timing of interventions is shown in Additional File 3B, with the highest impact of prenatal and postnatal counseling combined. Lay support had a significant impact, while education alone failed to achieve any statistical significance (Additional File 4B). The results were statistically significant for both individual counseling but not for group counseling (Additional File 5B) and also no difference according to the level of care (Additional File 6B) as all sub-group results were statistically insignificant.

#### Any breastfeeding at 4-6 weeks

There were 22 studies that included this outcome [44-46,48,49,51,54,55,58,59,61,63,75-84], of which one was from developing country [84]. There was a 10% statistically significant increase in any breastfeeding at 4-6 weeks, with a 14% increase in developing countries (based on one study) (Additional File 2C). The combination of prenatal and postnatal counseling did not have any impact on breastfeeding rate, according to the sub-group analyses, while prenatal and postnatal each alone had significant impacts (Additional File 3C). No other sub-group analyses were performed for this outcome.

#### Any breastfeeding at 6 months

Twenty studies included this outcome of any breastfeeding at 6 months [21,25,26,31,44,45,54,56,60,71-73,77-82,84-86], of which two were from developing countries [25,84]. There was a 12% statistically significant increase in any breastfeeding rates at 6 months (RR = 1.12; 95% CI: 1.01 – 1.24), while the results for developing and developing countries separately showed no significant impact (Additional File 2D). The impact was only significant if the promotion was given in prenatal and postnatal periods combined (Additional File 3D). No other sub-group analyses were performed for this outcome.

#### Recommendations for the LiST model

For the LiST model, we recommend estimates using the studies from developing countries. We recommend a 89% increase in rate of EBF at 4-6 weeks from promotion interventions (RR = 1.89; 95% CI: 1.50 – 2.37) and a 6 fold increase in rate of EBF at 6 months (RR = 12.14; 95% CI: 9.76 – 15.11). For EBFat 4-6 weeks, group counseling (RR = 1.67; 95% CI: 1.23 – 2.26) had a greater impact compared to individual counseling (RR = 1.38; 95% CI: 1.22 – 1.56), while for EBF at 6 months the effects of each were significant for individual counseling [RR 2.60, 95 % CI 1.13-5.96) but were insignificant for group counseling (RR = 2.03; 95% CI: 0.85 – 4.85).

## Discussion

This systematic review summarizes the effect of breastfeeding promotion interventions, including support and education, on exclusive and any breastfeeding rates at 4-6 weeks and at 6 months with recommendations for the LiST tool of estimates of EBF rates from developing country studies. The evidence grading for both EBF at 4-6 weeks and at 6 months was found to be ‘high’ based on directness, precision and consistency of the overall studies [33]. The results in both were statistically significant, with P-values of less than 0.1.

Our review has some limitations which stem primarily from the methodological shortcomings of the included studies. We found substantial clinical and methodological heterogeneity across studies because there was variability in interventions, definitions of outcomes, study design and risk of bias. This led to statistical heterogeneity and we performed different sub-group analyses to explore the cause of this heterogeneity. We did not, however, attempt sensitivity analyses. We finally used random models to redress heterogeneity that could not be explained. We included all the studies in our meta-analyses irrespective of their individual methodological quality. The risk of bias table of each study is given in Additional File 7. As can be seen from this table, quasi-randomized trials were also included where true randomization was not performed; blinding was not possible in a majority of studies which would lead to the possibility of observer bias and allocation concealment also remained unclear in most of these studies. The other main limitation was that relatively fewer studies were from developing world compared to developed countries.

There are other reviews on the subject. The Cochrane review on support for breastfeeding mothers [32] focuses on support interventions and has excluded studies that had an educational intent. It reports that with all forms of support, there was a significant 33% and non-significant 10% reduced risk of stopping EBFat 4-6 weeks and 6 months, respectively. Our review includes studies not restricted to the support, but includes all components of breastfeeding promotion, including education.

We have seen a greater increase in breastfeeding rate with promotion interventions in developing countries compared to developed countries. The first and foremost reason is that there was a difference in methodological conduct of studies from developing and developed countries. The other reasons may include that the baseline level of awareness and education among women of developing countries is less compared to those of developed nations. Besides, fewer mothers in developed countries would be receptive to breastfeeding promotion because of other factors like early employment, more ready availability of formula milk, and a different social milieu. The sub-group analyses on timing of breastfeeding show that prenatal counseling had greater impacts on breastfeeding rates at 4-6 weeks, while combined prenatal and postnatal promotion were important for breastfeeding rates at 6 months. For EBF at 4-6 weeks, most components of promotion interventions were important that included education alone, professional support alone, lay support alone and education plus professional support, while for EBF at 6 months, lay support and education plus professional support achieved statistical significance. For EBF at 4-6 weeks, group counseling had a better impact than individual counseling, while the results were insignificant for both individual and group counseling alone for EBF at six months. Community and facility promotion combined was found to be better than community or facility promotion alone.

While a 6 fold increase in EBF at 6 months is large, it is still well below recommendations; the intervention groups still failed to achieve high rates of EBF with the exception of Haider et al. [38]. This supports the observation that EBF is difficult and implies that it requires substantially more than education and support targeted solely at the mother to improve EBF rates in developing and developed countries.

## Conclusion

Breastfeeding promotion interventions significantly increased EBF rates at 4-6 weeks and at 6 months postpartum, with a greater effect in developing countries. Prenatal counseling was found to be of greater importance for breastfeeding at 4-6 weeks, while combined prenatal and postnatal counseling was of significant benefit for EBF at 6 months of age.

## Competing interests

We do not have any financial or non-financial competing interests for this review.

## Authors' contributions

Professor Zulfiqar A Bhutta developed the review parameters and obtained support. Dr Aamer Imdad and Dr Mohammad Yawar Yakoob undertook the literature search, data extraction and analysis under the supervision of Professor Bhutta. All contributed to the writing of the manuscript.

## Supplementary Material

Additional File 1 Characteristics of included studies. Click here for file

Additional File 2 A) Forest plot of the impact of breastfeeding promotion interventions on EBFrate at 4-6 weeks. B) Forest plot of the impact of breastfeeding promotion interventions on EBFrate at 6 months. C) Forest plot of the impact of breastfeeding promotion interventions on any breastfeeding rate at 4-6 weeks. D) Forest plot of the impact of breastfeeding promotion interventions on any breastfeeding rate at 6 months. Click here for file

Additional File 3 A) Sub-group analysis according to the timing of breastfeeding interventions on EBFrate at 4-6 weeks. B) Sub-group analysis according to the timing of breastfeeding interventions on EBFrate at 6 months. C) Sub-group analysis according to the timing of breastfeeding interventions on any breastfeeding rate at 4-6 weeks. D) Sub-group analysis according to the timing of breastfeeding interventions on any breastfeeding rate at 6 months. Click here for file

Additional File 4 A) Forest plot of sub-group analysis according to components of breastfeeding promotion interventions for EBFrates at 4-6 weeks. B) Forest plot of sub-group analysis according to components of breastfeeding promotion interventions for EBFrates at 6 month. Click here for file

Additional File 5 A) Forest plot of sub-group analysis for EBFat 4-6 weeks with respect to type of counseling. B) Forest plot of sub-group analysis for EBFat 6 months with respect to type of counseling. Click here for file

Additional File 6 A) Forest plot of sub-group analysis for EBFat 4-6 weeks with respect to the level of care at which the intervention was delivered. B) Forest plot of sub-group analysis for EBFat 6 months with respect to the level of care at which the intervention was delivered. Click here for file

Additional File 7 Risk of bias table of included studies. Click here for file
