# Supplementary material for: Effect of breastfeeding promotion interventions on breastfeeding rates, with special focus on developing countries
Source: BMC Public Health. 2011 Apr 13;11(Suppl 3):S24. doi: 10.1186/1471-2458-11-S3-S24 (PMC3231898; doi:10.1186/1471-2458-11-S3-S24)
Supplement: Additional File 2 — A) Forest plot of the impact of breastfeeding promotion interventions on EBFrate at 4-6 weeks. B) Forest plot of the impact of breastfeeding promotion interventions on EBFrate at 6 months. C) Forest plot of the impact of breastfeeding promotion interventions on any breastfeeding rate at 4-6 weeks. D) Forest plot of the impact of breastfeeding promotion interventions on any breastfeeding rate at 6 months. [file 1471-2458-11-S3-S24-S2.docx]

**Additional File 2A: Forest plot of the impact of breastfeeding promotion interventions on EBF rate at 4-6 weeks**

**Additional File 2B: Forest plot of the impact of breastfeeding promotion interventions on EBF rate at 6 months**

**Additional File 2C: Forest plot of the impact of breastfeeding promotion interventions on any breastfeeding rate at 4-6 weeks**

**Additional File 2D: Forest plot of the impact of breastfeeding promotion interventions on any breastfeeding rate at 6 months**
