# Supplementary material for: Effect of breastfeeding promotion interventions on breastfeeding rates, with special focus on developing countries
Source: BMC Public Health. 2011 Apr 13;11(Suppl 3):S24. doi: 10.1186/1471-2458-11-S3-S24 (PMC3231898; doi:10.1186/1471-2458-11-S3-S24)
Supplement: Additional File 3 — A) Sub-group analysis according to the timing of breastfeeding interventions on EBFrate at 4-6 weeks. B) Sub-group analysis according to the timing of breastfeeding interventions on EBFrate at 6 months. C) Sub-group analysis according to the timing of breastfeeding interventions on any breastfeeding rate at 4-6 weeks. D) Sub-group analysis according to the timing of breastfeeding interventions on any breastfeeding rate at 6 months. [file 1471-2458-11-S3-S24-S3.docx]

**Additional File 3A: Sub-group analysis according to the timing of breastfeeding interventions on EBF rate at 4-6 weeks**

**Additional File 3B: Sub-group analysis according to the timing of breastfeeding interventions on EBF rate at 6 months**

**Additional File 3C: Sub-group analysis according to the timing of breastfeeding interventions on any breastfeeding rate at 4-6 weeks**

**Additional File 3D: Sub-group analysis according to the timing of breastfeeding interventions on any breastfeeding rate at 6 months**
