# Supplementary material for: Effect of breastfeeding promotion interventions on breastfeeding rates, with special focus on developing countries
Source: BMC Public Health. 2011 Apr 13;11(Suppl 3):S24. doi: 10.1186/1471-2458-11-S3-S24 (PMC3231898; doi:10.1186/1471-2458-11-S3-S24)
Supplement: Additional File 4 — A) Forest plot of sub-group analysis according to components of breastfeeding promotion interventions for EBFrates at 4-6 weeks. B) Forest plot of sub-group analysis according to components of breastfeeding promotion interventions for EBFrates at 6 month. [file 1471-2458-11-S3-S24-S4.docx]

**Additional File 4A: Forest plot of sub-group analysis according to components of breastfeeding promotion interventions for EBF rates at 4-6 weeks**

**Additional File 4B: Forest plot of sub-group analysis according to components of breastfeeding promotion interventions for EBF rates at 6 months**
