# Supplementary material for: Effect of breastfeeding promotion interventions on breastfeeding rates, with special focus on developing countries
Source: BMC Public Health. 2011 Apr 13;11(Suppl 3):S24. doi: 10.1186/1471-2458-11-S3-S24 (PMC3231898; doi:10.1186/1471-2458-11-S3-S24)
Supplement: Additional File 5 — A) Forest plot of sub-group analysis for EBFat 4-6 weeks with respect to type of counseling. B) Forest plot of sub-group analysis for EBFat 6 months with respect to type of counseling. [file 1471-2458-11-S3-S24-S5.docx]

**Additional File 5A: Forest plot of sub-group analysis for EBF at 4-6 weeks with respect to type of counseling**

**Additional File 5B: Forest plot of sub-group analysis for EBF at 6 months with respect to type of counseling**
